# Silver Diamine Fluoride vs Atraumatic Restoration for Managing Dental Caries in Schools: A Cluster Randomized Clinical Trial

**DOI:** 10.1001/jamanetworkopen.2025.13826

**Published:** 2025-06-09

**Authors:** Ryan Richard Ruff, Aditi Ashish Gawande, Qianhui Xu, Tamarinda Barry Godín

**Affiliations:** 1 Department of Epidemiology & Health Promotion, New York University, New York, New York

## Abstract

**Importance:**

Dental caries is a pervasive and inequitable chronic disease stemming from a lack of access to preventive and therapeutic care. Minimally invasive interventions may be provided in schools to treat caries in children.

**Objective:**

To compare the effectiveness of silver diamine fluoride (SDF) with atraumatic restorative treatment (ART) in the control of dental caries among US schoolchildren.

**Design, Setting, and Participants:**

The CariedAway study was a cluster randomized clinical trial conducted from February 1, 2019, to June 1, 2023, in 48 primary schools in New York City. Participants were followed up for up to 4 years. Schools with a student population of at least 50% Black and/or Hispanic or Latino students and 80% receiving free or reduced-cost lunch were eligible. Within enrolled schools, any child with parental informed consent was eligible. Treatment was provided biannually. Analysis was restricted to children aged 5 to 13 years who completed at least 1 follow-up observation and had at least 1 tooth surface with dental caries.

**Interventions:**

Participants were randomized at the school level to receive SDF or ART.

**Main Outcomes and Measures:**

Any surface lesion with an International Caries Detection and Assessment System score of 5 or 6 was recorded as caries. The primary outcome was the number of carious surfaces that had a recurrence of caries. Analysis was performed on an intent-to-treat basis.

**Results:**

Of the 17 741 children eligible, 7418 were randomized (mean [SD] age at baseline, 7.6 [1.9] years; 4006 girls [54.0%]), and 1668 were analyzed (mean [SD] age at baseline, 6.8 [1.5] years; 881 girls [52.8%]; 861 in the SDF group and 807 in the ART group). The total surface-level failure in the SDF group was 38.3% (2167 of 5651 carious surfaces) compared with 45.5% (2116 of 4647) in the ART group. There were 2167 surface failures observed among SDF participants over 1372 person-years compared with 2116 ART surface failures over 1291 person-years (incidence rate ratio, 0.96 [95% CI, 0.91-1.02]). At the person level, 45.5% of SDF recipients (392 of 861) experienced at least 1 surface failure compared with 53.3% of ART recipients (430 of 807; odds ratio, 0.51 [95% CI, 0.39-0.66]). There were no significant differences in the risk of recurrent surface failure between treatments (hazard ratio, 0.92 [95% CI, 0.82-1.04]).

**Conclusions and Relevance:**

In this study of treatments for caries, similar failures in surface control were observed among children receiving SDF or ART. These results support the use of secondary preventive therapies for caries in schools.

**Trial Registration:**

ClinicalTrials.gov Identifier: NCT03442309

## Key Points

**Question:**

How effective is silver diamine fluoride (SDF) compared with atraumatic restorative treatment (ART) in managing dental caries in schools?

**Findings:**

In this 4-year cluster randomized clinical trial, the total surface-level failure of SDF-treated tooth surfaces with caries was 38.3%, compared with 45.5% in the ART group, but this difference was not significant; a total of 45.5% of participants receiving SDF experienced at least 1 control failure, compared with 53.3% of ART recipients, which was significant. There were no differences in the risk of recurrent surface failures.

**Meaning:**

This study suggests that SDF, used as part of school-based dental care, can effectively control caries.

## Teaser

This cluster randomized clinical trial compares the effectiveness of silver diamine fluoride with atraumatic restorative treatment in the control of dental caries over 4 years among US schoolchildren.

## Introduction

Dental caries (tooth decay) is a worldwide public health crisis, affecting billions of children and adults^1^ who often lack access to effective preventive or therapeutic care.^2^ It is also highly inequitable, as those from low-income families or racial and ethnic minority families shoulder a disproportionate burden of disease.^3^ Untreated caries may increase the risk of systemic noncommunicable diseases, including cancer, diabetes, cardiovascular diseases, and neurodegenerative conditions.^4^ Caries also affects child development, reducing educational performance^5^ and oral health–related quality of life,^6^ and is responsible for more than 30 million hours of lost seat time in schools per year.^7^

Integrating preventive and therapeutic dental services into schools can increase access to care and reduce the risk of caries and may improve educational performance.^8^ The US Department of Health and Human Services’ Community Preventive Services Task Force recommends school sealant programs to prevent dental caries,^9^ and the Centers for Disease Control and Prevention funds school-based sealant programs in multiple states. These programs are both clinically effective and cost-effective.^10,11^ However, managing existing caries among children who are unlikely to seek out traditional, office-based care remains a critical issue in dental public health.

Silver diamine fluoride (SDF) and glass ionomer atraumatic restorative treatment (ART, or atraumatic restorations) are minimally invasive interventions that can effectively arrest or control caries, and they are included on the World Health Organizations’ Model List of Essential Medicines. Silver diamine fluoride is a topical solution that inhibits the growth of cariogenic bacteria and contributes to the remineralization of enamel and dentin caries,^12^ whereas ART removes decalcified tissue with hand instruments before applying adhesive fillings to restore the cavity.^13^ Silver diamine fluoride is estimated to arrest anywhere from 47% to 90% of caries lesions after 1 application^14^ and is considered to be a practical, affordable approach to community caries prevention, particularly in low-socioeconomic areas.^15^ In contrast, the failure rate for single- and multiple-surface ARTs in primary molars after 2 years is estimated to be 6% and 35%, respectively,^16^ although this may be outperformed by conventional restorations.^17^ Like SDF, prior studies of ART in community settings concluded that it is acceptable and effective in socioeconomically deprived groups.^18^

The CariedAway study^19^ was a cluster randomized clinical trial of the use of SDF and ART in schools. In this study, we estimated the effects of SDF compared with ART for caries control over 4 years. A secondary objective was to determine whether posterior application of SDF resulted in subsequent anterior caries control.

## Methods

CariedAway was a longitudinal, cluster randomized, pragmatic clinical trial conducted (in real-world settings) from February 1, 2019, to June 1, 2023, in eligible primary schools in New York City.^19^ The study received institutional review board approval from the New York University School of Medicine and is registered at ClinicalTrials.gov (NCT03442309). Parents provided written informed consent, and students provided verbal assent. The trial protocol is included in Supplement 1. Trial results are reported following the Consolidated Standards of Reporting Trials (CONSORT) reporting guideline for randomized trials. The trial is completed and is not open to new enrollment.

The primary objectives of the CariedAway trial were to determine whether SDF was noninferior compared with ART in the 2-year arrest of caries and noninferior compared with glass ionomer cement (GIC) sealants in the 4-year prevention of new caries.^20,21^ The present analysis for 4-year recurrent arrest was not an original objective of the study. Trial participants included in the present analysis were only those enrolled who had dental caries (eTable in Supplement 2).

### Participants

Any primary school in the New York City geographic area with a student population consisting of at least 50% Black and/or Hispanic or Latino students and at least 80% receiving free or reduced-cost lunch was eligible to participate. These inclusion criteria were used as those schools typically have the highest burden of disease in the New York metropolitan area. Paper and electronic consent forms were sent to the parent or guardian of every child enrolled in participating schools. Any child with signed parental informed consent and child assent was enrolled in the study. Although care was provided to any child meeting these criteria, participation in the trial was restricted to those aged 5 to 13 years. Parents or guardians were not present for care as it was provided during the school day. Participants did not receive any costs for care.

### Randomization

Clusters (participating schools) were block randomized to either the experimental or active control condition using a random number generator performed by R.R.R. and verified by T.B.G. All children in a school received the same treatment.

### Interventions, Procedures, and Masking

For untreated caries, study participants received either SDF followed by fluoride varnish (to mask the bitter aftertaste of SDF) or GIC ART followed by fluoride varnish. In the experimental group, a 38% concentration SDF solution (2.24 F-ion mg/dose) was applied to cavitated lesions on asymptomatic posterior teeth, including primary molars and permanent molars and premolars. The SDF application used a microapplicator after quadrant isolation with gauze and cotton rolls. Silver diamine fluoride was applied for a minimum of 30 seconds, and treated sites were then air dried for a minimum of 60 seconds. In the active control group, quadrant isolation was achieved with gauze and cotton rolls. Glass ionomer cement ART was then applied on the same asymptomatic posterior teeth using the finger-sweep technique, ensuring that the material was in direct contact with the walls of the lesion and closed margins were achieved. Excess material (eg, flash) was removed with disposable dental explorers and cotton tip applicators. Separately, dental sealants were applied to sound dentition in the active control but were not included in analysis. Treatments were provided in a private, dedicated room in a school using portable dental mirrors, explorers, and dental chairs with a frontal light source. All treatments were administered by either dental hygienists (SDF and ART) or registered nurses (SDF) under patient-specific standing orders signed by a supervising dentist. Examination and treatment were provided biannually, except for disruptions in care due to COVID-19. Silver diamine fluoride was applied at each observational visit regardless of surface caries status, whereas ART was only reapplied if failed. As a pragmatic clinical trial, masking was not performed.

### Data Collection and Diagnosis Protocol

Race and ethnicity, age, and sex of participating children were self-reported by parents on informed consent documents. Racial and ethnic categories were those required to be collected by any school-based health program operating in New York City; they were assessed in the study as the focus was children of minority racial and ethnic groups, primarily Hispanic or Latino and Black. Prior to treatment, all children received an oral examination at each study observation. All tooth surfaces were recorded for sound, decayed, missing, or filled status. Missing teeth were classified as “missing” in accordance with dental age. Caries diagnosis was performed after a visual-tactile dental screening according to the International Caries Detection and Assessment System (ICDAS) adapted criteria in epidemiology and clinical research settings.^22^ Any lesion with an ICDAS score of 5 (distinct cavity with visible dentin) or 6 (extensive or more than half the surface distinct cavity with visible dentin) was recorded as caries. At each observational visit, the first instance of dental caries was recorded and treated following the described clinical protocols.

### Examiners and Standardization

Treatments were provided by dental hygienists (ART and SDF) or registered nurses (SDF) with the assistance of dental assistants and under the supervision of a licensed pediatric dentist. Clinicians received 70 hours of didactic and practical training, including clinical presentations and hands-on application of techniques using typodonts. Instructor feedback was provided and peer learning sessions were conducted to reinforce understanding. Examiner training sessions involved live participants presenting with carious lesions of varying severity. Findings were systematically reviewed by a senior examiner (the study supervising licensed dentist [T.B.G.]), with repeated assessments conducted until interexaminer agreement was achieved. Standardization was performed annually for all personnel.

### Outcome Definition

Outcomes were measured at the individual level. Primary outcomes for this analysis were the number of controlled and failed surfaces for each study participant. Caries control was determined if a carious surface (mesial, occlusal, distal, buccal, or lingual) on treatable teeth (permanent molars, premolars, or primary molars) received the assigned treatment and did not present with recurrent caries at any subsequent observation over the course of the study. Any caries recurrence was classified as control failure, and any surfaces on which the first observation of caries occurred at the student’s last study visit were removed from analysis. Although the total maximum observation for failure was 4 years, the actual time until failure from initial treatment was recorded, allowing for each participant to contribute data for as long as they were enrolled in the trial. In addition, any controlled surface that was exfoliated and replaced by carious permanent dentition was not considered control failure, nor was any treated surface that later presented with a restoration placed by an outside clinician (eg, dentist). Based on these outcomes, the per-person control proportion (*C*) was then computed as the number of controlled surfaces divided by the total number of treated surfaces, and the per-person failure proportion was computed as 1 − *C*.

### Power

As caries control over 4 years was not a primary end point of the CariedAway trial, an a priori power analysis for this outcome was not performed. Power analyses for primary end points, accounting for intracluster correlations, were previously reported.^20,21^

### Statistical Analysis

Descriptive statistics for the full study enrollment and for participants with at least 1 follow-up observation and at least 1 carious surface were computed overall and by treatment group (mean [SD] values and frequencies). We calculated the total number of controlled and failed surfaces observed for each study participant over a maximum of 4 years of follow-up, estimating the incidence rate ratio of failure. Controlled and failed surfaces were also determined by sex and race and ethnicity. To account for potential within-school correlation, we subsequently analyzed person-level failure using generalized linear mixed-effects models with a random intercept for school. To determine the effect of individual-level disease severity on failure rates, we included a variable for the per-person number of carious surfaces and an interaction effect between disease severity and treatment. We then considered the possibility of recurrent surface failure using frailty models with variables for treatment type and selected participant demographic variables. A random effect was included for dependence among recurrent event times. For simplicity, we first analyzed data ignoring higher-level school clustering but then used nested frailty models to include random effects at individual and school levels. Finally, to determine the association between posterior SDF application and anterior surface caries control, we restricted the sample to participants assigned to the SDF group who presented with both posterior and anterior decay on any surface at baseline. We then calculated the number of decayed anterior surfaces at baseline and the number of controlled surfaces at first follow-up and measured the strength of the association using the Pearson correlation. Statistical analysis followed intent to treat and was conducted in R, version 4.3.3 (R Project for Statistical Computing). Statistical significance was determined at *P* < .05, and all tests were 2-sided.

## Results

A total of 7418 participants (mean [SD] age at baseline, 7.6 [1.9] years; 4006 girls [54.0%] and 3412 boys [46.0%]; 125 Asian students [1.7%], 1246 Black students [16.8%], 3648 Hispanic or Latino students [49.2%], 153 White students [2.1%], 114 students of >1 race [1.5%], and 90 students of other race or ethnicity [1.2%]) in 48 schools were enrolled in the CariedAway trial from February 1, 2019, to June 1, 2023, consisting of 3739 (50.4% [24 schools]) randomized to the SDF group and 3679 (49.6% [24 schools]) randomized to the ART group (Table 1; Figure). The overall baseline decay prevalence was 26.7% (1980 of 7418). The intraclass correlation for carious dentition clustering within schools was 0.01. For the analytic sample (participants with at least 1 follow-up observation and presenting with at least 1 surface-level carious lesion prior to their last treatment visit), 1668 students (mean [SD] age at baseline, 6.8 [1.5] years; 881 girls [52.8%] and 787 boys [47.2%]; 39 Asian students [2.3%], 343 Black students [20.6%], 916 Hispanic or Latino students [54.9%], 41 White students [2.5%], 21 students of >1 race [1.3%], and 37 students of other race or ethnicity [2.2%]) met the inclusion criteria; 861 (51.6%) were randomized to the SDF group, and 807 (48.4%) were randomized to the ART group. A total of 1140 of the 1668 students in the analytic sample (68.4%) had initial caries at baseline, while the remaining students developed caries over the course of the study. The analytic sample was equally distributed with respect to baseline decay, sex, age, and race and ethnicity. No adverse events were reported.

**Table 1.** Patient Characteristics for Total Enrollment and Analytic Sample

Characteristic	Participants, No. (%)		
	Overall	SDF	ART
Participants enrolled	7418 (100)	3739 (50.4)	3679 (49.6)
Baseline decay	1980 (26.7)	1016 (27.2)	964 (26.2)
Sex			
Male	3412 (46.0)	1785 (47.7)	1627 (44.2)
Female	4006 (54.0)	1954 (52.3)	2052 (55.8)
Race and ethnicity			
Asian	125 (1.7)	88 (2.4)	37 (1.0)
Black	1246 (16.8)	650 (17.4)	596 (16.2)
Hispanic or Latino	3648 (49.2)	1766 (47.2)	1882 (51.2)
White	153 (2.1)	86 (2.3)	67 (1.8)
More than 1	114 (1.5)	67 (1.8)	47 (1.3)
Other^a^	90 (1.2)	56 (1.5)	34 (0.9)
Unreported	2042 (27.5)	1026 (27.4)	1016 (27.6)
Age at baseline, mean (SD), y	7.6 (1.9)	7.5 (1.9)	7.6 (1.9)
Participants analyzed^b^	1668 (100)	861 (51.6)	807 (48.4)
Baseline decay	1140 (68.4)	584 (67.8)	556 (68.9)
Sex			
Male	787 (47.2)	414 (48.1)	373 (46.2)
Female	881 (52.8)	447 (51.9)	434 (53.8)
Race and ethnicity			
Asian	39 (2.3)	28 (3.3)	11 (1.4)
Black	343 (20.6)	184 (21.4)	159 (19.7)
Hispanic or Latino	916 (54.9)	455 (52.9)	461 (57.1)
White	41 (2.5)	24 (2.8)	17 (2.1)
More than 1	21 (1.3)	14 (1.6)	7 (0.9)
Other^a^	37 (2.2)	25 (2.9)	12 (1.5)
Unreported	271 (16.3)	131 (15.2)	140 (17.4)
Age at baseline, mean (SD), y	6.8 (1.5)	6.8 (1.6)	6.8 (1.4)

Abbreviations: ART, atraumatic restorative treatment; SDF, silver diamine fluoride.

^a^ The “Other” category for race and ethnicity reported by caregivers included American Native and Hawaiian or Pacific Islander.

^b^ The CariedAway trial included objectives for the prevention of caries from sound dentition and the control of caries in diseased dentition. This reflects the difference in enrolled vs analyzed participants, as not all children in the study had caries to control.

**Figure.**
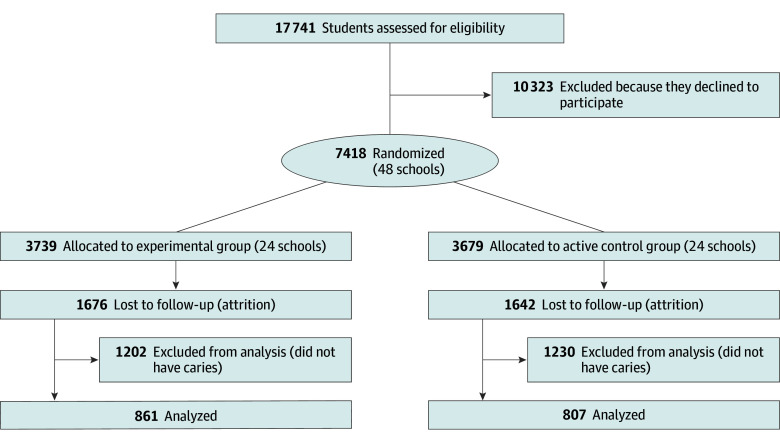
Trial Enrollment Flowchart

A total of 1066 of the 1668 participants in the analytic sample (63.9%) had between 1 and 3 surfaces with decay; 314 participants had 1 carious surface, 494 had 2 carious surfaces, and 258 had 3 carious surfaces. Estimates of the crude incidence rates show that there were 5651 total carious surfaces that received SDF (Table 2). Of these surfaces, 2167 presented with subsequent decay, for a surface-level failure rate of 38.3%. In the ART group, 4647 total carious surfaces were treated, of which 2116 presented with later decay, for a failure rate of 45.5%. The person-level failure rates in both groups were slightly higher, estimated at 45.5% for the SDF group (392 of 861) and 53.3% for the ART group (430 of 807). When considering the total time of observation (1372 person-years in the SDF group and 1291 person-years in the ART group), the incidence rate ratio was 0.96 (95% CI, 0.91-1.02; *P* = .23). Similar rates were found across treatments by sex, but there were significant differences between treatments in select racial and ethnic groups.

**Table 2.** Unadjusted IRRs for Failures in Carious Surfaces Treated With SDF and ART^a^

Characteristic	SDF				ART				Unadjusted IRR (95% CI)
	No. of total surfaces treated	No. of total surfaces failed	No. of children with surface failure	Total No. of days until failure	No. of total surfaces treated	No. of total surfaces failed	No. of children with surface failure	Total No. of days until failure	
Analytic sample	5651	2167	392	500 759	4647	2116	430	471 227	0.96 (0.91-1.02)
Sex									
Male	2793	1069	204	250 588	2217	1025	199	226 223	0.94 (0.86-1.03)
Female	2858	1098	188	250 171	2430	1091	231	245 004	0.99 (0.91-1.07)
Race and ethnicity									
Asian	160	61	11	17 158	59	12	4	6789	1.95 (1.05-3.62)
Black	1338	576	85	101 764	944	467	92	98 797	1.20 (1.06-1.35)
Hispanic or Latino	2927	1140	218	288 339	2704	1217	241	278 208	0.90 (0.83-0.98)
White	107	32	6	8954	67	30	8	11 860	1.42 (0.86-2.34)
More than 1	127	45	9	9670	25	8	2	519	0.43 (0.20-0.82)
Other^b^	190	92	18	9884	119	57	8	7219	1.19 (0.86-1.66)
Unreported	802	221	45	64 990	729	325	75	67 835	0.71 (0.60-0.84)

Abbreviations: ART, atraumatic restorative treatment; IRR, incidence rate ratio; SDF, silver diamine fluoride.

^a^ The total number of failed surfaces and the total number of days until failure were used to calculate IRRs.

^b^ The “Other” category for race and ethnicity reported by caregivers included American Native and Hawaiian or Pacific Islander.

After adjusting for the potential clustering within schools, the odds of person-level failure was significantly reduced among children receiving SDF compared with ART (odds ratio [OR], 0.51 [95% CI, 0.39-0.66]) (Table 3). Disease severity was also significantly associated with control failure, with each additional decayed surface increasing the odds of failure by approximately 16% (OR, 1.16 [95% CI, 1.13-1.19]). However, there was no significant interaction between treatment and disease severity (OR, 0.96 [95% CI, 0.91-1.01]).

**Table 3.** Individual-Level Failure by Treatment and Disease Severity^a^

Characteristic	Odds ratio (95% CI)	*P* value
SDF vs ART	0.51 (0.39-0.66)	<.001
Disease severity^b^	1.16 (1.13-1.19)	<.001
Sex (male)	1.12 (0.91-1.39)	.28
Race and ethnicity		
Asian	0.71 (0.35-1.45)	.35
Black	1.02 (0.78-1.35)	.87
Hispanic or Latino	1 [Reference]	NA
White	0.62 (0.31-1.24)	.18
More than 1	0.99 (0.39-2.53)	.99
Other^c^	2.07 (0.97-4.42)	.06
Unreported	0.83 (0.61-1.12)	.22
Multiplicative model		
Severity × SDF	0.96 (0.91-1.01)	.13

Abbreviations: ART, atraumatic restorative treatment; NA, not applicable; SDF, silver diamine fluoride.

^a^ Generalized linear mixed-effects models with random intercepts. A multiplicative model was adjusted for all covariates displayed in additive models.

^b^ Disease severity was defined as the total number of teeth with caries.

^c^ The “Other” category for race and ethnicity reported by caregivers included American Native and Hawaiian or Pacific Islander.

For recurrent surface failure, similar to unadjusted rate ratios, children receiving SDF did not experience a statistically significant reduction in the risk of recurrent surface failure vs ART (hazard ratio [HR], 0.92 [95% CI, 0.82-1.04]) (Table 4). In nested models adjusting for visit and school, effects were not appreciably different (HR, 0.82 [95% CI, 0.49-1.15]), and there were no significant associations with sex and race and ethnicity.

**Table 4.** Hazard Ratios of Recurrent Surface-Level Failure Among Trial Participants^a^

Characteristic	Hazard ratio (95% CI)
SDF (vs ART)	0.92 (0.82-1.04)
Male	1.05 (0.93-1.19)
Race and ethnicity	
Asian	0.96 (0.60-1.55)
Black	1.11 (0.95-1.30)
Hispanic or Latino	1 [Reference]
White	0.69 (0.46-1.03)
More than 1	1.54 (0.90-2.65)
Other^b^	1.11 (0.75-1.66)
Unreported	1.09 (0.90-1.31)

Abbreviations: ART, atraumatic restorative treatment; SDF, silver diamine fluoride.

^a^ Frailty model analysis of recurrent surface failure, estimating hazard ratios.

^b^ The “Other” category for race and ethnicity reported by caregivers included American Native and Hawaiian or Pacific Islander.

For the potential for anterior control, there were 462 anterior surfaces with decay at baseline across 108 participants who had both posterior and anterior decay and received SDF on posterior carious lesions. Among these participants, 920 posterior teeth received SDF. At first follow-up observation, 6 anterior surfaces were arrested (0.01%) across 2 students. There was no correlation between anterior surface arrest and the number of posterior teeth treated with SDF (*r* = −0.001; *P* = .99).

## Discussion

The CariedAway study was a school-based clinical trial of minimally invasive treatments for caries management. More than 1600 children received either SDF or ART on more than 10 000 carious tooth surfaces. In the SDF group, the surface-level failure rate was 38.3%, and the participant-level failure rate was 45.5%; in the ART group, the surface-level failure rate was 45.5%, and the participant-level failure rate was 53.3%. Silver diamine fluoride recipients outperformed ART recipients at the individual level, but there were no significant differences in the risk of recurrent failure in adjusted analyses.

Although the use of SDF and ART in US schools is limited, a prior study of school-based application of ART reported reductions in the per-visit risk of caries by approximately 20% among children with untreated decay.^23^ Another study found that a single application of SDF or ART halted caries progression among 56% and 46%, respectively, of high-risk schoolchildren 2 years after initial treatment.^21^ Our results support similar conclusions for repeat application after 4 years. In addition, previous estimates were computed at the individual level, whereas our data are based on more robust surface-level failure rates. Results also corroborate international studies, in which SDF has been provided as part of preschool oral health programs, demonstrating both clinical effectiveness and a high rate of acceptance among parents.^15^ Similarly, school-based ART programs have demonstrated 30-month restoration survival rates ranging from 33% to 79% in Hong Kong,^24^ 5-month success rates of 82% in Brazil,^25^ and 12-month single-surface success rates of 97% in the Republic of Kosovo.^26^ Outside of school settings, both SDF and ART were found to be effective in a community trial in Australian Aboriginal children,^27^ and a mobile dental program operating in underserved Mexican communities reported a 66% retention rate for ART after 2 years.^28^

The effectiveness of using SDF and ART for caries management in schools may be modified by the severity of disease, such as the size and depth of the carious lesion. Differences in arrest attributed to treatment may also be affected by disease severity.^29^ The American Academy of Pediatric Dentistry (AAPD) reports a wide range of caries arrest rates for SDF, with effectiveness varying by cavity size and tooth location,^14^ and notes that the arresting capacity of SDF may be greater in less-severe lesions. A systematic review of ART among children estimated a 71% success rate after 1 year and a 67% success rate after 2 years, but the success of multiple-surface restorations was significantly lower than single-surface ones.^28^ Accordingly, the AAPD policy reports that single-surface applications have higher survival than multiple-surface restorations.^30^ We present evidence that the risk of failure increases with each additional diseased surface present, suggesting possible limitations of school-based approaches for children with considerable unmet needs. However, there were no differences in the effect of disease severity across treatment groups. In addition, this measure of severity reflects only an increased number of opportunities for failure, as we were unable to stratify by ICDAS score in our analysis. Further study of whether failure risk depends on multiple-surface decay vs single-surface decay in these settings would be useful.

Frequency of application is also likely to affect caries control. A review of SDF studies suggests semiannual application^31^ to mitigate the reduction in effectiveness for caries arrest that may occur over time, while the AAPD Clinical Practice Guidelines recommend a single application with follow-up after 2 to 4 weeks, reapplying where indicated. Other approaches use more intensive early application, such as weekly application over a 3-week period, in an attempt to enhance arresting effects.^32^ Similar to other school-based dental programs,^33^ the clinical protocols for CariedAway stipulated semiannual application of both SDF and ART (if not retained, in the case of ART). However, this initial schedule was disrupted due to school closings related to the COVID-19 pandemic. As a result, the elapsed time between treatments had considerable variability for enrolled participants. Our results suggest that observed failure rates with repeat application are similar to those after a single application.^21^ It may be the case that the effectiveness of SDF and ART is reduced somewhat in pragmatic settings, such as school-based caries prevention. Nevertheless, the overall success rate is encouraging for community caries management.

The direct effects of SDF have received considerable study^12,34,35^; the mechanistic action of SDF includes reducing the growth of cariogenic bacteria through the antibacterial effect of silver and also by promoting the remineralization of enamel and dentin caries. Silver ions in SDF are thought to prevent bacterial aggregation through reaction with bacterial cellular surface proteins,^35^ harden soft carious lesions through reaction with phosphate or chloride ions resulting in the formation of silver salts (eg, silver chloride), and increase the alkalinity of the environment through the formation of ammonium compounds hypothesized to have an acid-buffering effect. However, the potential indirect effects of application on adjacent caries activity have not been meaningfully examined. Silver diamine fluoride application in CariedAway was restricted to posterior dentition to mitigate any negative effect that SDF staining might have on facial aesthetics and oral health–related quality of life. We subsequently explored whether posterior application controlled anterior lesions and whether there was any dose-response relationship. Our data do not indicate any such association, and we observed no correlation between the number of posterior surfaces treated and any subsequent anterior arrest.

### Limitations

There are a number of study limitations. Due to the gaps in care because of COVID-19, exfoliation of primary dentition in the intervening period may have occurred. As a result, follow-up for affected tooth surfaces would stop if recorded as missing and then restart for newly erupted permanent dentition if later diagnosed to be carious. In addition, it is possible that if a study participant were to have visited an outside dental clinician, the examining dentist may have put a permanent restoration in place of any dentition previously treated with SDF or ART. Although we consider this possibility to be unlikely given the target population enrolled, any observation of a filling on a surface previously treated with either SDF or ART would not be recorded as decayed, which would potentially inflate the success percentage of treatments.

## Conclusions

In this school-based cluster randomized clinical trial, the longitudinal control of surface-level dental caries was not significantly different when comparing SDF with ART. However, at the individual level, those receiving SDF had significantly higher control of caries. Incorporating minimally invasive techniques in schools can help improve access to critical preventive oral medicine and decrease health inequities.

## Notes

Supplement 1.
Trial Protocol and Statistical Analysis Plan

Supplement 2. **eTable.** Differences in Enrolled Versus Analyzed Sample

Supplement 3.
Data Sharing Statement
