# Supplementary material for: Silver Diamine Fluoride vs Atraumatic Restoration for Managing Dental Caries in Schools: A Cluster Randomized Clinical Trial
Source: JAMA Netw Open. 2025 Jun 9;8(6):e2513826. doi: 10.1001/jamanetworkopen.2025.13826 (PMC12150187; doi:10.1001/jamanetworkopen.2025.13826)
Supplement: Supplement 2. — eTable. Differences in Enrolled Versus Analyzed Sample [file jamanetwopen-e2513826-s002.pdf]

Supplementary Online Content

Ruff RR, Gawande AA, Xu Q, Barry Godín T. Silver diamine fluoride vs atraumatic restoration for managing dental caries in schools: a cluster randomized clinical trial. *JAMA Netw Open.* 2025;8(6):e2513826. doi:10.1001/jamanetworkopen.2025.13826

eTable. Differences in Enrolled Versus Analyzed Sample

This supplementary material has been provided by the authors to give readers additional information about their work.

© 2025 Ruff RR et al. *JAMA Network Open.*

eTable. Differences in Enrolled Versus Analyzed Sample

	Overall		SDF		ART	
	N	%	N	%	N	%
**Participants analyzed**	1668	100	861	51.62	807	48.38
Baseline decay	1140	68.35	584	67.83	556	68.9
Sex (male)	787	47.18	414	48.08	373	46.22
Females	881	52.82	447	51.92	434	53.78
Race/Ethnicity						
Asian	39	2.34	28	3.25	11	1.36
Black	343	20.56	184	21.37	159	19.7
Hispanic/Latino	916	54.92	455	52.85	461	57.13
More than one	21	1.26	14	1.63	7	0.87
Other	37	2.22	25	2.9	12	1.49
Unreported	271	16.25	131	15.21	140	17.35
White	41	2.46	24	2.79	17	2.11
**Participants not analyzed**	5750	100	2878	50.05	2872	49.95
Baseline decay	840	14.61	432	15.01	408	14.21
Sex (male)	2625	45.65	1371	47.64	1254	43.66
Females	3125	54.35	1507	52.36	1618	56.34
Race/Ethnicity						
Asian	86	1.50	60	2.08	26	0.91
Black	903	15.70	466	16.19	437	15.22
Hispanic/Latino	2732	47.51	1311	45.55	1421	49.48
More than one	93	1.62	53	1.84	40	1.39
Other	53	0.92	31	1.08	22	0.77
Unreported	1771	30.80	895	31.10	876	30.50
White	112	1.95	62	2.15	50	1.74

© 2025 Ruff RR et al. *JAMA Network Open.*
