# Supplementary material for: Silver Diamine Fluoride vs Atraumatic Restoration for Managing Dental Caries in Schools: A Cluster Randomized Clinical Trial
Source: JAMA Netw Open. 2025 Jun 9;8(6):e2513826. doi: 10.1001/jamanetworkopen.2025.13826 (PMC12150187; doi:10.1001/jamanetworkopen.2025.13826)
Supplement: Supplement 3. — Data Sharing Statement [file jamanetwopen-e2513826-s003.pdf]

Data Sharing Statement

Ruff. Silver Diamine Fluoride vs Atraumatic Restoration for Managing Dental Caries in Schools. *JAMA Netw Open*. Published June 06, 2025. doi:10.1001/jamanetworkopen.2025.13826

Data
Additional Information: www.clinicaltrials.gov, NCT03442309
Data available: Yes
Data types: Deidentified participant data, Data dictionary
How to access data: Data will be made available upon request and approval of a data use agreement and analytic plan.
When available: With publication

Supporting Documents
Document types: None

Additional Information
Who can access the data: researchers whose proposed use of the data has been approved
Types of analyses: De-identified data
Mechanisms of data availability: After approval of a proposal
